# The Role of High-Sensitivity *C*-Reactive Protein in Activities of Daily Living Among Middle-Aged and Older Adults: A Prospective Cohort Study

**DOI:** 10.3390/nu17101732

**Published:** 2025-05-20

**Authors:** Shu-Min Lai, Ling Kuang, Xu-Lian Tang, Cheng-Shen Qiu, Hong-Xuan Huang, Dan-Qing Liao, Hong-Min Li, Li-Ying Du, Zhi-Yuan Xiong, Bing-Yun Zhang, Hao-Jie Chen, Zhi-Hao Li

**Affiliations:** Department of Epidemiology, School of Public Health, Southern Medical University, Guangzhou 510515, China; laishumin0119@163.com (S.-M.L.); kuangling22@163.com (L.K.); tangxulianpm@163.com (X.-L.T.); qiuchsh2021@gmail.com (C.-S.Q.); huanghongxuan0113@163.com (H.-X.H.); liaodq0926@163.com (D.-Q.L.); hongminli2209@163.com (H.-M.L.); duliying02@163.com (L.-Y.D.); xiongzhy7@163.com (Z.-Y.X.); zhangbingyun00@163.com (B.-Y.Z.); chenhjie2024@163.com (H.-J.C.)

**Keywords:** high-sensitivity *C*-reactive protein, ADL disability, inflammation

## Abstract

**Background:** The association between high-sensitivity *C*-reactive protein (hsCRP) and activities of daily living (ADL) disability remains unclear. Our study aimed to comprehensively explore the relationship between hsCRP concentrations and the risk of ADL disability, while also identifying potential modifiers of this association in middle-aged and older adults. **Methods:** We conducted a prospective study involving 16,342 participants aged 50 years and older (mean age: 64 ± 10 years) from the Health and Retirement Study. To investigate the longitudinal association between hsCRP and the risk of ADL disability, we employed Cox proportional hazard regression models, adjusting for a wide range of potential confounders. Subgroups analyses were further conducted to examine interactions across factors such as gender, age, body mass index, smoking status, and drinking status. **Results:** This study involved a follow-up of 125,858 person-years (median of 8 years; interquartile range: 4–12 years), revealing a total of 4579 incidents of ADL disability. The highest hsCRP concentration was significantly associated with ADL disability after adjustment for covariates (hazard ratio [HR] = 1.25; confidence interval [CI] = 1.14–1.36). The associations between hsCRP and the risk of ADL disability seemed to be somewhat stronger among individuals aged < 65 years and with a BMI ≥ 30 kg/m^2^ (both *p* for interaction < 0.05). **Conclusions:** Our findings indicated that elevated hsCRP concentrations are associated with an increased risk of ADL disability in middle-aged and older adults. HsCRP appears to serve as a biomarker for ADL disability, particularly among individuals with obesity and middle-aged adults.

## 1. Introduction

High-sensitivity *C*-reactive protein (hsCRP) is an acute-phase protein synthesized by the liver during inflammatory responses [1]. It has emerged as a reliable biomarker for systemic inflammation due to its relatively stable concentrations, cost-effective detection, and high sensitivity [2]. Previous research has indicated that hsCRP is associated with various adverse health outcomes, including an increased risk of mortality, cardiovascular disease, and higher rates of disability [3,4,5,6].

Physical functional impairment is one of the most common health problems among the elderly in the United States, imposing a considerable burden on both individuals and society [7]. Specifically, activities of daily living (ADL) disability, as the core manifestation of physical functional impairment, has become a crucial factor affecting the quality of life of the elderly [8]. ADL disability is defined as the inability of an individual to independently perform basic tasks in daily life, including essential activities such as dressing, eating, doing laundry, and walking [9]. Current studies mainly focus on the clinical state of ADL disability, which is irreversible and leaves little opportunity for interventions to delay the process; it is valuable to explore the predictable factors to delay the onset and reduce the severity of ADL disability. Recent studies have demonstrated that elevated hsCRP concentrations are associated with the pathogenesis of sarcopenia, frailty, and functional limitations, which are critical determinants of ADL disability [6,10,11]. Despite several investigations into the association between hsCRP and the risk of ADL disability, the findings remain inconsistent [12,13,14,15,16]. For instance, a cross-sectional study conducted among elderly individuals in Chinese communities revealed an inverse association between serum hsCRP and ADL performance (n < 3500) [12]. In addition, a cohort study conducted among a community-residing population aged 70 and older explored the relationship between hsCRP and ADL decline, showing that elevated hsCRP levels were associated with an increased risk of ADL disability (n = 624) [13]. Another study involving 2610 men and women aged 65 and older identified a link between hsCRP levels and ADL disability; however, this association was not significant in women [17]. Furthermore, prior studies have demonstrated a significant positive link between obesity and inflammatory markers like hsCRP [18,19]. Research also indicates that the body’s inflammatory response capability declines with age, leading to notable differences in hsCRP levels across age groups [20]. However, there are certain limitations in the current related research. Most studies adopt a cross-sectional research design, and the sample sizes are generally small. Several confounding factors, such as chronic diseases and lifestyles choices, have not been adequately controlled, which are highly likely to interfere with the determination of the relationship between hsCRP and ADL performance. Additionally, there is a lack of understanding regarding whether the associations between hsCRP and ADL disability differ based on the body mass index (BMI), gender, or age subgroups within population studies.

Therefore, we conducted a prospective cohort study using data from the 2006–2016 Health and Retirement Study (HRS) community cohort. By controlling for confounding factors from multiple aspects, we aimed to explore the association between hsCRP and the risk of ADL disability in individuals aged 50 and above. Additionally, we performed a series of subgroup analyses to explore the modifying effects of various factors, such as age, BMI, smoking status, and drinking status, among others.

## 2. Methods and Materials

### 2.1. Study Design and Population

This study used data from the HRS, a nationally representative, community-based, prospective cohort study sample of the United States population aged 50 and older. Information on the study participants and its design has been reported before [19]. In brief, interviews with the participants were conducted in 1992 and then repeated at two-year intervals. From 1994 to 2016, six new groups of participants were incorporated in stages. Commencing in 2006, as part of the HRS, an upgraded face-to-face interview, which involved a biomarker assessment, was carried out. In all the HRS research activities, the Declaration of Helsinki guidelines were adhered to, and procedures were approved by the University of Michigan Health Sciences/Behavioral Sciences Institutional Review Board (IRB) (Protocol: HUM00061128). Before enrolling any participants, written informed consent was collected.

**Figure 1:**
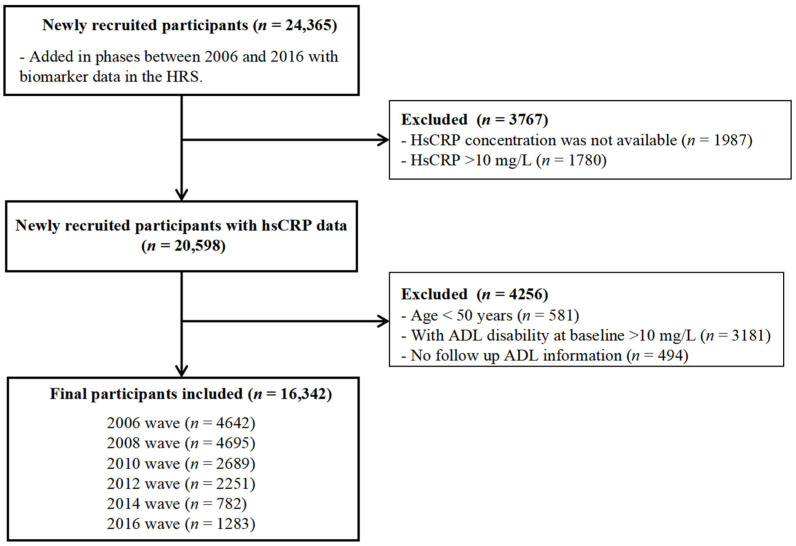
Flowchart of the sample selection process.

In the present study, we used data from the 2006 to 2016 waves of the HRS. The participants who had missing data on hsCRP and those with hsCRP concentrations of greater than 10 were excluded due to association with acute illness and major trauma; furthermore, individuals with limitations in any of the six ADLs at the baseline were not included in the study. A total of 16,342 individuals, comprising 7178 men and 9164 women, were deemed eligible for inclusion in the study. Figure 1 presents a flowchart of participant enrollment.

### 2.2. Measurement of hsCRP

The plasma hsCRP concentration was measured in serum using a latex-particle-enhanced immunoturbidimetric assay kit. The minimum detectable value for the hsCRP concentration stood at 0.035 mg/L. The within-assay variability was 8.1%, and the between-assay variability was 11.0%. All the participants were categorized into four groups according to quartiles. These quartiles were defined as follows: quartile 1 (Q1), with values of less than 0.61 mg/L; quartile 2 (Q2), ranging from 0.61 to 1.29 mg/L; quartile 3 (Q3), from 1.30 to 2.73 mg/L; and quartile 4 (Q4), with values of greater than 2.73 mg/L.

### 2.3. Assessment of ADL Disability

The ability to perform ADLs was ascertained by a questionnaire that asked the participants of the HRS whether they had any difficulty with the following six tasks: (1) dressing, including putting on shoes and socks; (2) eating, such as cutting your food; (3) using the toilet, such as getting up; (4) bathing or showering; (5) getting into or out of bed; and (6) walking across a room. The respondents were asked to exclude any difficulties expected to last less than 3 months. Following previous studies [21,22], we dichotomized ADL disability into “no limitation = 0” or “at least one limitation = 1”. All the respondents were free of ADL disability at baseline. The participants were defined as having “ADL disability” if one or more limitations emerged during the follow-up period.

### 2.4. Covariates

We selected a wide range of potential confounders for inclusion in this study by drawing on evidence from prior epidemiological investigations [23,24]. Covariates encompassed sociodemographic details such as age, sex, and race/ethnicity. Lifestyle-related covariates, such as the body mass index (BMI), smoking status, and drinking status, were included. Clinical measures included the concentrations of total cholesterol (TC), high-density lipoprotein cholesterol (HDL-C), and hemoglobin A1c (HbA1c). Additionally, the score from the 8-item Center for Epidemiologic Studies Depression Scale (CES-D 8) was included. Moreover, chronic conditions, like hypertension, diabetes, cancer, and arthritis, were among the covariates. The BMI was calculated from body weight and height measurements calculated by trained personnel. A BMI < 18.5 kg/m^2^ was defined as low, a BMI between 18.5 and 24.9 kg/m^2^ was considered normal, a BMI between 25.0 and 29.9 kg/m^2^ was classified as overweight, and a BMI ≥ 30.0 kg/m^2^ was regarded as obese, according to the World Health Organization [25]. The entirety of the data associated with covariates was procured from the structured questionnaire and the biochemistry examinations that were executed at the baseline. The original contributions presented in the study are publicly available. These data can be found here (http://hrsonline.isr.umich.edu, accessed on 25 September 2024).

### 2.5. Statistical Analysis

Descriptive data were used to summarize the participant characteristics, with continuous variables presented as the means (standard deviation, SD) and categorical variables presented as counts (percentage) stratified by hsCRP quartiles. Cox proportional hazard models were applied to estimate hazard ratios (HRs), and 95% confidence intervals (95% CIs) were applied to estimate the risk of ADL disability according to the hsCRP quartiles, using the lowest quartile as the reference. We also evaluated the HRs for the risk of ADL disability for each 1 mg/L increase in hsCRP. To evaluate potential nonlinear associations, restricted cubic splines (RCSs) with 3 knots were incorporated into the Cox models, and the linearity assumption was tested via likelihood ratio tests comparing the models with linear and spline terms. We used two Cox hazard regression models, which were adjusted for different sets of variables and evaluated using Schoenfeld residual plots. Model 1 tested the association between hsCRP and the risk of ADL disability controlled for age and sex; Model 2 further adjusted for ethnicity, BMI, smoking status, drinking status, regular exercise, HDL-C, TC, HbA1c, CES-D 8 scores, hypertension, diabetes, cancer, and arthritis. To increase the statistical power, we utilized the multiple imputation through chained equations to fill in the missing covariate data [26]. The effect modifications of the associations between each 1 mg/L increase in hsCRP and the risk of ADL disability by sex (men or women), age (<65 or ≥65 years), BMI (obese or non-obese), current smoking status, and current drinking status were assessed by calculating and comparing likelihood ratios for their statistical fit with interaction terms in the multivariable adjusted models. To address any potential Type I error from multiple testing, we adjusted *p*-values for interaction effects in the subgroup analyses using two methods: (1) Bonferroni correction (dividing *α* = 0.05 by 2, the number of tests); (2) False Discovery Rate (FDR) control via Benjamini-Hochberg method.

To ensure the robustness of our findings, we performed a series of sensitivity analyses, including excluding all the participants who died within the 2 years prior to follow-up; stratifying individuals by tertiles, quintiles, and clinically relevant categories of hsCRP [27]. All the analyses were conducted using R 4.3.2, and a significant difference was defined as *p* < 0.05.

## 3. Results

### 3.1. Baseline Characteristics

Table 1 provides the details of the participants’ characteristics, with the participants stratified by the quartiles of hsCRP measured at baseline. We followed a cohort of 16,342 participants, with a median age of 64 years old (standard deviation: 10 years), of whom 56% were women. The median value of hsCRP was 1.27 mg/L. The participants with higher hsCRP levels were more likely to be women, of white ethnicity, and current drinkers and to have a higher BMI (all *p* < 0.05). Compared to the participants without limitation, those who with ADL disability were older, predominantly female, engaged in less regular exercise, and had higher incidences of hypertension, diabetes, cancer, and arthritis (all *p* < 0.05, Table S1).

### 3.2. HsCRP and the Risk of ADL Disability

**Figure 2:**
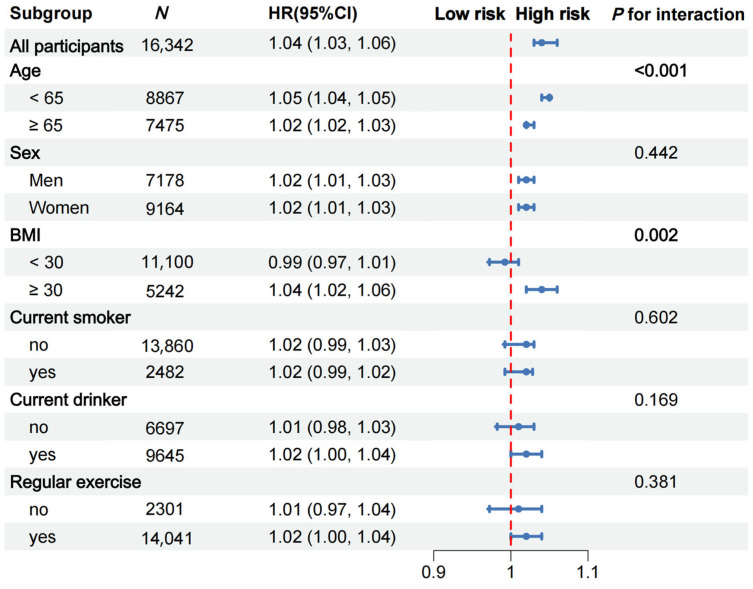
Subgroup analyses for the hazard ratios (HRs) of ADL disability. Adjusted for age, sex, race, current smoking status, current drinking status, regular exercise, BMI, TC, HDL-C, HbA1c, CES-D 8 score, hypertension, diabetes, cancer, and arthritis.

**Figure 3:**
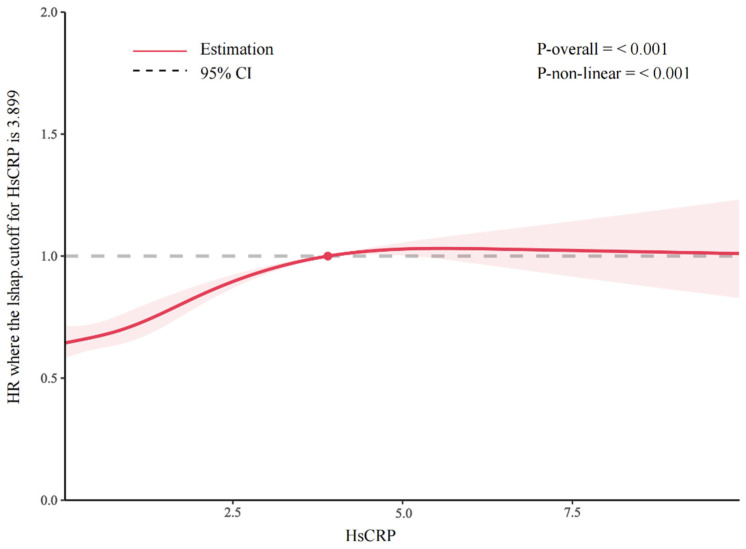
Hazard ratios (HRs) of hsCRP concentration on ADL disability. The results are from the restricted cubic spline Cox proportional hazard regression model.

The total number of person-years comprising the follow-up was 125,858, and the median follow-up time was 8 years (interquartile range [IQR] = 4–12 years); we recorded 4579 incidents of ADL disability events. The multivariable-adjusted HRs with 95% CIs for ADL disability from the lowest to the highest quartiles of the hsCRP level were 1 (reference), 1.08 (95% CI, 0.99–1.17), 1.24 (95% CI, 1.14–1.35), and 1.52 (95% CI, 1.40–1.65) (*p* for trend < 0.001) (Table 2). Further analysis demonstrated that each 1 mg/L increment of hsCRP was significantly associated with a 3% increased risk of ADL disability, with an HR (95% CI) of 1.03 (1.01–1.04) (Figure 2). Additionally, a nonlinear and positive association between the hsCRP concentration and the risk of incident ADL disability events was identified using a restricted cubic spline regression (*p* for nonlinearity < 0.001) (Figure 3).

### 3.3. Subgroup Analyses

Subgroup and interaction analyses were performed to identify potential modifying factors. The positive associations between hsCRP and the risk of ADL disability were stronger among those aged < 65 years (*p* for interaction < 0.001, Figure 2) and those with a BMI ≥ 30 kg/m^2^ (*p* for interaction = 0.002; Figure 2). After considering multiple comparisons, the interactions between hsCRP and age, as well as between the CRP and BMI groups, remain significant (*p* < 0.05) under both the Bonferroni correction and the FDR control (Table S6). However, no significant interaction effects were found for the other four predefined subgroups (all *p* for interactions > 0.05).

### 3.4. Sensitivity Analyses

The associations between hsCRP and ADL disability remained consistent when participants who died within 2 years of follow-up were excluded (Table S2). Moreover, when categorizing hsCRP levels as tertiles or quintiles, the results were similar (Tables S3 and S4), and when using clinically relevant categories (Table S5) based on hsCRP, the results remained materially unchanged.

## 4. Discussion

In this prospective cohort study, we observed that elevated levels of hsCRP were associated with a higher incidence of ADL disability among middle-aged and elderly individuals. Even after thorough adjustment for sociodemographic factors, metabolic biomarkers, lifestyle choices, and chronic disease history, these associations persisted as robust, suggesting that hsCRP levels may play an independent role in the decline of ADL performance. Moreover, our findings highlight that the risk of hsCRP contributing to ADL disability is significantly heightened among obese individuals and those aged under 65 years.

Several studies have assessed the relationship between hsCRP and ADL disability events, and most indicate that individuals who have higher hsCRP levels are likely to have deficits in ADL performance [12,13,14,15,16,28]. Moreover, a prospective study conducted in a Japanese population indicates that hsCRP is favorably associated with physical performance, even within a very low range (<1.0 mg/L) [15]. In addition, these associations have also been found in special populations. In chronic kidney disease patients, higher hsCRP levels increase the risk of ADL disability [29]. Among stroke survivors, baseline hsCRP levels predict ADL recovery [6]. In elderly patients with cognitive impairment, elevated hsCRP is linked to faster ADL decline. By overcoming the sample size and design limitations of previous studies, our study also found a significant association between elevated hsCRP levels and the risk of ADL disability using a large, community-based cohort of middle-aged and older adults. These findings affirm the significant utility of hsCRP, a highly sensitive and readily measurable biomarker, in the early prediction and detection of ADL disability [30]. By identifying individuals with elevated hsCRP levels at an early stage, healthcare professionals can implement evidence-based, proactive interventions. These interventions may encompass personalized exercise regimens designed to enhance physical function, targeted nutritional counseling aimed at modulating inflammatory pathways, and weight management programs aimed at halting the progression of functional impairment and potentially deferring or even preventing the onset of ADL disability.

Moreover, our study found that the association between hsCRP and ADL disability was more pronounced in individuals with obesity. Prior studies have indicated that obesity is known to relate to the length of a life with a disability before death or an increase in the severity of the disability occurs [31,32]. Similarly, a study noted that obese individuals with elevated inflammatory levels experience a faster decline in muscle strength and physical function compared to non-obese individuals, thereby increasing the risk of impaired physical performance [33]. A potential explanation for this is that excessive energy intake may lead the body into a pro-inflammatory state, amplifying the adverse effects of inflammation on physical function and significantly raising the risk of ADL disability [34]. While some studies suggest a higher risk of ADL disability among women, possibly due to relatively lower physical fitness compared to men [35], this pattern was not evident in our study. This discrepancy may be attributed to our focus on individuals aged 50 and above, who are typically in the perimenopausal stage, where female sex hormones may have less influence on ADL disability outcomes [36].

The mechanisms by which hsCRP is associated with the development of functional limitations are not fully understood. They may be mediated through several biological pathways [37,38,39]. First, chronic inflammation, as indicated by elevated hsCRP, can lead to muscle degradation through increased protein catabolism and oxidative stress [39,40]. This process may result in sarcopenia, a condition characterized by the loss of muscle mass and strength, which is a critical determinant of ADL disability among older adults. Second, hsCRP is also linked to the development of various chronic diseases, such as diabetes, cardiovascular disease, and cognitive decline [41]. The inflammation-induced hepatic acute-phase response prioritizes the synthesis of inflammatory proteins such as hsCRP, while inhibiting the production of nutrition-related proteins like albumin and prealbumin, thereby exacerbating malnutrition [42,43]. Inadequate protein intake leads to insufficient nutrient intake in older adults, which in turn activates inflammatory pathways. This process impairs muscle repair capacity, thereby accelerating functional decline [44]. Despite these proposed mechanisms, further research is needed to fully elucidate the complex interplay between hsCRP and functional decline. Future studies should prioritize exploring interventions like dietary changes and lifestyle modifications, which can safeguard muscle function and uphold seniors’ independence. Such efforts are crucial for improving their quality of life and reducing the healthcare burden tied to ADL disability.

## 5. Strengths and Limitations

This investigation offers several methodological strengths, including a population-based design with adequate power to detect moderate effect sizes. The incorporation of longitudinal biomarker data enhances clinical translatability while controlling for key confounders. Nevertheless, our findings should be considered in the context of several limitations. First, our reliance on single-timepoint hsCRP measurements may not fully capture chronic inflammatory exposure. Second, our study did not consider other potential confounding factors that could influence both hsCRP concentrations and the risk of ADL disability. Third, there may have been a selection bias relating to our participants, as healthier individuals were more likely to be followed-up for a longer period, which could have introduced a healthy survivor bias. Finally, although our study involved a relatively large sample size, it was conducted in a specific geographic region, which may have introduced a regional bias and limits the generalizability of the results to other populations.

## 6. Conclusions

Our study demonstrates a significant association between elevated hsCRP and the risk of ADL disability among middle-aged and older individuals. The findings highlight the importance of considering inflammation as a potential risk factor for functional decline and suggest that targeted interventions to reduce inflammation may help preserve ADL function in vulnerable populations.

## Figures and Tables

**Table 1:** Characteristics of study participants.

Characteristics	Overall	HsCRP Concentrations Quartiles (mg/L)			
		Q1 (<0.61)	Q2 (0.61–1.29)	Q3 (1.30–2.73)	Q4 (>2.73)
No. of participants	16,342	4071	4110	4078	4083
Age, years	64.48 (10.20)	65.40 (10.45)	65.50 (10.29)	64.27 (10.08)	62.72 (9.72)
Women (%)	9164 (56%)	2087 (51%)	2119 (52%)	2346 (58%)	2612 (64%)
Race (%)					
White	12,244 (75%)	3237 (80%)	3247 (79%)	3051 (75%)	2709 (66%)
Black	2765 (17%)	521 (13%)	528 (13%)	695 (17%)	1021 (25%)
Other	1333 (8%)	313 (7%)	335 (8%)	332 (8%)	353 (8%)
BMI, kg/m^2^ (%)					
<30	11,100 (68%)	3399 (83%)	3021 (74%)	2638 (65%)	2042 (50%)
≥30	5242 (32%)	672 (17%)	1089 (26%)	1440 (35%)	2041 (50%)
Current smoker (%)	2482 (15%)	479 (12%)	529 (13%)	695 (17%)	779 (19%)
Current drinker (%)	9645 (59%)	2541 (62%)	2465 (60%)	2401 (59%)	2238 (55%)
Regular exercise (%)	14,041 (86%)	3633 (89%)	3589 (87%)	3485 (85%)	3334 (82%)
HDL-C, mg/dL	63.72 (21.20)	64.48(21.56)	64.80 (21.37)	63.03 (20.93)	63.58 (20.92)
HbA1c, mg/dL	5.79 (0.91)	5.67 (0.75)	5.75 (0.85)	5.80 (0.90)	5.93 (1.07)
TC, mg/dL	225.19 (66.22)	212.91 (59.38)	221.16 (62.91)	228.71 (67.71)	238.24 (71.75)
CES-D 8 score	1.24 (1.80)	1.11 (1.68)	1.14 (1.74)	1.27 (1.79)	1.43 (1.95)
Hypertension (%)	8818 (54%)	1876 (46%)	2205 (54%)	2246 (55%)	2491 (61%)
Diabetes (%)	3127 (19%)	653 (16%)	742 (18%)	767 (19%)	965 (24%)
Cancer (%)	2016 (12%)	519 (13%)	498 (12%)	495 (12%)	504 (12%)
Arthritis (%)	8202 (50%)	1948 (48%)	2060 (50%)	2110 (52%)	2084 (51%)

Continuous variables are shown as mean (SD); categorical variables are shown as the number of cases (%).

**Table 2:** HRs (95% CI) for ADL disability stratified by baseline hsCRP concentration quartiles.

HsCRP	ADL Disability			
	Model 1 ^a^	*p*	Model 2 ^b^	*p*
No. of participants	16,342			
No. of events	4579			
Q1	1.00 (reference)	-	1.00 (reference)	-
Q2	1.08 (0.99, 1.17)	0.086	1.02 (0.94, 1.11)	0.597
Q3	1.24 (1.14, 1.35)	<0.001	1.10 (1.01, 1.19)	0.022
Q4	1.52 (1.40, 1.65)	<0.001	1.23 (1.13, 1.34)	<0.001
*p* for trend	<0.001		<0.001	

^a^ Model 1: adjusted for age and sex. ^b^ Model 2: adjusted for age, sex, race, current smoking status, current drinking status, regular exercise, BMI, TC, HDL-C, HbA1c, CES-D 8 score, hypertension, diabetes, cancer, and arthritis.

## Data Availability

The original contributions presented in the study are publicly available. These data can be found here (http://hrsonline.isr.umich.edu, accessed on 25 September 2024).

## Supplementary Materials

The following supporting information can be downloaded at https://www.mdpi.com/article/10.3390/nu17101732/s1. Table S1. Demographic and clinical characteristics of study participants with varying status of ADL disability; Table S2. HRs (95% CI) for ADL disability stratified by hsCRP quartiles excluding participants who died within 2 years of follow-up; Table S3. HRs (95% CI) of ADL disability by tertiles of high-sensitivity C-reactive protein; Table S4. HRs (95% CI) of ADL disability by quintiles of high-sensitivity *C*-reactive protein; Table S5. HRs (95% CI) of ADL disability according to clinical categories of high-sensitivity *C*-reactive protein; Table S6. Subgroup analyses for the HRs (95% CI) of ADL disability for each 1 mg/L increase in high-sensitivity *C*-reactive protein.

## Abbreviations

HsCRP	high-sensitivity *C*-reactive protein
ADL	activities of daily living
BMI	body mass index
HR	hazard ratio
CI	confidence interval
HRS	Health and Retirement Study
DBS	Dried blood spot
TC	total cholesterol
HDL-C	high-density lipoprotein cholesterol
HbA1c	hemoglobin A1c
CES-D 8	8-item Center for Epidemiologic Studies Depression Scale
SDs	standard deviations
RCSs	restricted cubic splines
