# Supplementary material for: The Role of High-Sensitivity C-Reactive Protein in Activities of Daily Living Among Middle-Aged and Older Adults: A Prospective Cohort Study
Source: Nutrients. 2025 May 20;17(10):1732. doi: 10.3390/nu17101732 (PMC12113845; doi:10.3390/nu17101732)
Supplement: Supplementary file 1 [file nutrients-17-01732-s001.zip › nutrients-3632848-supplementary.pdf]

Supplement

Table of Contents

Table S1. Demographic and clinical characteristics of study participants with varying status of ADL disability....................1

Table S2. HRs (95% CI) for ADL disability stratified by hsCRP quartiles excluding participants who died within 2 years of follow-up....................2

Table S3. HRs (95% CI) of ADL disability by tertiles of high-sensitivity C-reactive protein....................3

Table S4. HRs (95% CI) of ADL disability by quintiles of high-sensitivity C-reactive protein....................4

Table S5. HRs (95% CI) of ADL disability according to clinical categories of high-sensitivity C-reactive protein....................5

Table S6. Subgroup analyses for the HRs (95% CI) of ADL disability for each 1 mg/L increase in high-sensitivity C-reactive protein....................6

Table S1. Demographic and clinical characteristics of study participants with varying status of ADL disability

Characteristics	Overall	No limitation	ADL disability	*P* value
No. of participants	16,342	11,769	4,579	
HsCRP concentrations	1.99 (1.98)	1.97 (1.98)	2.04 (1.99)	0.010
Age, years	64.48 (10.20)	62.99 (9.69)	68.33 (10.50)	<0.001
Women (%)	9,164 (56%)	6,425 (55%)	2,739 (60%)	<0.001
Race (%)				<0.001
White	12,244 (75%)	8,780 (75%)	3,464 (76%)	
Black	2,765 (17%)	1,948 (17%)	817 (18%)	
Other	1,333 (8%)	1,035 (8.8%)	298 (6.5%)	
BMI, kg/m^2 (%)	28.23 (5.49)	27.99 (5.30)	28.84 (5.90)	<0.001
Current smoker (%)	2,482 (15%)	7,323 (62%)	2,322 (51%)	<0.001
Current drinker (%)	9,645 (59%)	7,323 (62%)	2,322 (51%)	<0.001
Regular exercise (%)	14,041 (86%)	10,448 (89%)	3,593 (78%)	<0.001
HDL-C, mg/dL	63.72(21.20)	64.80 (21.70)	60.97 (19.62)	<0.001
HbA1c, mg/dL	5.79 (0.91)	5.73 (0.84)	5.93 (1.04)	<0.001
TC, mg/dL	225.19 (66.22)	230.50 (67.46)	211.59 (60.90)	<0.001
CES-D 8 score	1.24 (1.80)	1.06 (1.63)	1.69 (2.09)	<0.001
Hypertension (%)	8,818 (54%)	5,904 (50%)	2,914 (64%)	<0.001
Diabetes (%)	3,127 (19%)	1,979 (17%)	1,148 (25%)	<0.001
Cancer (%)	2,016 (12%)	1,330 (11%)	686 (15%)	<0.001
Arthritis (%)	8,202 (50%)	5,121 (44%)	3,081 (67%)	<0.001

[1]Mean (SD); n (%)

[2]Wilcoxon rank sum test; Pearson's Chi-squared test; Fisher's exact test

BMI, body mass index; CES-D 8, 8-question Center for Epidemiologic Studies Depression Scale; HbA1c, hemoglobin A1c; HDL-C, high-density lipoprotein cholesterol; TC, total cholesterol.

Table S2. HRs (95% CI) for ADL disability stratified by hsCRP quartiles excluding participants who died within 2 years of follow-up

**HsCRP quartiles**	**ADL disability**			
	**Model 1[a]**	***P***	**Model 2[b]**	***P***
No. of participants		15843		
No. of events		4579		
Q1	1.00 (reference)	-	1.00 (reference)	-
Q2	1.07 (0.98, 1.16)	0.101	1.02 (0.93, 1.10)	0.658
Q3	**1.25 (1.15, 1.35)**	**<0.001**	**1.10 (1.01, 1.20)**	**0.020**
Q4	**1.54 (1.42, 1.67)**	**<0.001**	**1.25 (1.14, 1.36)**	**<0.001**
P for trend	**< 0.001**		**< 0.001**	

[a] Model 1: adjusted for age and sex

[b] Model 2: adjusted for age, sex, race, BMI, current smoking status, current drink status, regular exercise, TC, HDL-C, HbA1c, CES-D 8 score, hypertension, diabetes, cancer and arthritis.

Table S3. HRs (95% CI) of ADL disability by tertiles of high-sensitivity C-reactive protein

HsCRP tertiles	ADL disability			
	**Model 1[a]**	***P***	**Model 2[b]**	***P***
No. of participants		16342		
No. of events		4579		
Q1 (<0.61 mg/L)	1.00 (reference)	-	1.00 (reference)	-
Q2 (0.61-2.73 mg/L)	**1.14 (1.05, 1.22)**	**<0.001**	1.04 (0.97, 1.12)	0.210
Q3 (> 2.73 mg/L)	**1.41 (1.31, 1.51)**	**<0.001**	**1.16 (1.08, 1.25)**	**<0.001**
P for trend	**< 0.001**		**< 0.001**	

[a] Model 1: adjusted for age and sex

[b] Model 2: adjusted for age, sex, race, BMI, current smoking status, current drink status, regular exercise, TC, HDL-C, HbA1c, CES-D 8 score, hypertension, diabetes, cancer and arthritis.

Table S4. HRs (95% CI) of ADL disability by quintiles of high-sensitivity C-reactive protein

HsCRP quintiles	ADL disability			
	**Model 1[a]**	***P***	**Model 2[b]**	***P***
No. of participants	16342			
No. of events	4579			
Q1 (<0.50 mg/L)	1.00 (reference)	-	1.00 (reference)	-
Q2 (0.50-0.95 mg/L)	1.06 (0.96, 1.16)	0.219	1.03 (0.94, 1.13)	0.472
Q3 (0.96-1.72 mg/L)	1.09 (0.99, 1.20)	0.060	0.99 (0.90, 1.09)	0.937
Q4 (1.73-3.23 mg/L)	**1.30 (1.18, 1.42)**	**<0.001**	**1.12 (1.02, 1.23)**	**0.013**
Q5 (>3.23 mg/L)	**1.52 (1.38, 1.66)**	**<0.001**	**1.21 (1.10, 1.33)**	**<0.001**
P for trend	**< 0.001**		**< 0.001**	

[a] Model 1: adjusted for age and sex

[b] Model 2: adjusted for age, sex, race, BMI, current smoking status, current drink status, regular exercise, TC, HDL-C, HbA1c, CES-D 8 score, hypertension, diabetes, cancer and arthritis.

Table S5. HRs (95% CI) of ADL disability according to clinical categories of high-sensitivity C-reactive protein

**HsCRP levels**	**ADL disability**			
	**Model 1[a]**	***P***	**Model 2[b]**	***P***
No. of participants		16342		
No. of events		4579		
<1.00 mg/L	1.00 (reference)	-	1.00 (reference)	-
1.00-3.00 mg/L	**1.17 (1.09, 1.25)**	<0.001	1.04 (0.97, 1.12)	0.173
> 3.00 mg/L	**1.46 (1.35, 1.58)**	**<0.001**	**1.19 (1.09, 1.29)**	**<0.001**
P for trend	**< 0.001**		**< 0.001**	

[a] Model 1: adjusted for age and sex

[b] Model 2: adjusted for age, sex, race, BMI, current smoking status, current drink status, regular exercise, TC, HDL-C, HbA1c, CES-D 8 score, hypertension, diabetes, cancer and arthritis.

Table S6. Subgroup analyses for the HRs (95% CI) of ADL disability for each 1 mg/L increase in high-sensitivity C-reactive protein

Subgroups	HR	*P* for interaction	$P_{\text{Bonferroni}}$	P_{FDR}
Age		**< 0.001**	**0.002**	**0.002**
< 65	1.05 (1.04, 1.05)			
≥ 65	1.02 (1.02, 1.03)			
Sex		0.442	1.000	0.870
Men	1.02 (1,01, 1.03)			
Women	1.02 (1.01, 1.03)			
BMI		**0.002**	**0.035**	**0.017**
< 30	0.99 (0.97, 1.01)			
≥ 30	1.04 (1.02, 1.06)			
Current smoker		0.602	1.000	0.870
no	1.02 (0.99, 1.03)			
yes	1.02 (0.99, 1.02)			
Current drinker		0.169	0.676	0.225
no	1.01 (0.98, 1.03)			
yes	1.02 (1.00, 1.04)			
Regular exercise		0.381	1.000	0.551
no	1.01 (0.97, 1.04)			
yes	1.02 (1,00, 1.04)			
